# A Simple, Test-Based Method to Control the Overestimation Bias in the Analysis of Potential Prognostic Tumour Markers

**DOI:** 10.3390/cancers15041188

**Published:** 2023-02-13

**Authors:** Marzia Ognibene, Annalisa Pezzolo, Roberto Cavanna, Davide Cangelosi, Stefania Sorrentino, Stefano Parodi

**Affiliations:** 1 U.O.C. Genetica Medica, IRCCS Istituto Giannina Gaslini, 16147 Genova, Italy; 2 IRCCS Istituto Giannina Gaslini, 16147 Genova, Italy; 3 Unità di Bioinformatica Clinica, IRCCS Istituto Giannina Gaslini, 16147 Genova, Italy; 4 U.O.C. Oncologia Pediatrica, IRCCS Istituto Giannina Gaslini, 16147 Genova, Italy; 5 Direzione Scientifica, IRCCS Istituto Giannina Gaslini, 16147 Genova, Italy

**Keywords:** tumour markers, hazard ratio, Cox model, survival analysis, overestimation bias, neuroblastoma, *E2F1*

## Abstract

**Simple Summary:**

Studies aimed at assessing the potential prognostic role of gene expression profiles in cancer patients often employ running procedures to select optimal cut-offs for the identification of groups with a poorer outcome. The corresponding hazard ratio (*HR*) is the most frequently used measure of association between gene expression and patient survival, but it is prone to an overestimation bias. If rare diseases are investigated in the absence of an external cohort, it is difficult to control this lack of accuracy. We propose a simple, test-based method to obtain the *HR*, adjusted for the overestimation bias. Validation using both simulated data and gene expression profiles from two publicly available data sets is provided. Furthermore, we show that the proposed method is able to identify a new gene with potential oncogenic activity in the reanalysis of a data set including 134 patients affected by Stage 4S neuroblastoma.

**Abstract:**

The early evaluation of prognostic tumour markers is commonly performed by comparing the survival of two groups of patients identified on the basis of a cut-off value. The corresponding hazard ratio (*HR*) is usually estimated, representing a measure of the relative risk between patients with marker values above and below the cut-off. A posteriori methods identifying an optimal cut-off are appropriate when the functional form of the relation between the marker distribution and patient survival is unknown, but they are prone to an overestimation bias. In the presence of a small sample size, which is typical of rare diseases, the external validation sets are hardly available and internal cross-validation could be unfeasible. We describe a new method to obtain an unbiased estimate of the *HR* at an optimal cut-off, exploiting the simple relation between the *HR* and the associated *p*-value estimated by a random permutation analysis. We validate the method on both simulated data and set of gene expression profiles from two large, publicly available data sets. Furthermore, a reanalysis of a previously published study, which included 134 Stage 4S neuroblastoma patients, allowed for the identification of *E2F1* as a new gene with potential oncogenic activity. This finding was confirmed by an immunofluorescence analysis on an independent cohort.

## 1. Introduction

The early evaluation of potential tumour markers for prognostic applications is usually carried out by comparing the survival of two groups of patients identified on the basis of a threshold value. A cut-off selected a priori can be used for such a purpose, based on the percentiles of the observed marker distribution as median, tertile, or quartile values [1]. In most cases, the functional form of the relation between the marker distribution and the patients’ survival is unknown, and a posteriori methods for the identification of an optimal cut-off could be more appropriate. For instance, a running method is usually applied that recursively splits the data set on the basis of each observed marker value. For each cut-off, some measure of distance between the survival probabilities in the two groups is calculated and the cut-off corresponding to the highest value is retained. Common measures include survival estimates via the Kaplan–Meier method, the associated log-rank test statistic, and the hazard ratio (*HR*) obtained by the corresponding Cox regression model [2]. In any case, results of the analysis may be prone to an overestimation bias that can be controlled by a validation procedure on an independent cohort [1,3,4]. In the absence of an external cohort, an internal cross-validation can be applied, randomly splitting the available data into (at least) one training and one test set [4]. However, in the presence of a small sample size, the cross-validation procedure cannot be applied. A *p*-value, adjusted for the overestimation bias, can be obtained to test the null hypothesis of no association between the marker levels and the patients’ survival by a random permutation analysis. The distribution of the corresponding test statistic is obtained under the null hypothesis by recursively swapping the follow-up times and the corresponding outcome indicators in couples of patients randomly selected in the data set [4,5]. The proportion of simulated values that are higher than the observed values provides an estimate of the corresponding *p*-value but does not remove the overestimation bias from the *HR*. The purpose of this study is to illustrate a simple, test-based method to obtain *HR* estimates that are adjusted for the overestimation bias. An example of its application will be provided by a reanalysis of data from a previously published study involving 134 patients with Stage 4S neuroblastoma (NB) [6].

Section 2 describes the new proposed method used to obtain unbiased *HR* estimates. Section 3 shows the results of the statistical validation, which used data from two publicly available data sets. Section 4 reports the results of the validation on the simulated data. Section 5 provides an application to a real data set, evaluating the potential prognostic role of three gene expression profiles in a reanalysis of a previously published study. Section 6 provides the biological validation of the results reported in Section 5, using information from an independent cohort. Section 7 includes the discussion of the obtained results.

## 2. Adjusting the Hazard Ratio for the Overestimation Bias

Let *T* be the (likely biased) test statistic calculated for the comparison of two groups identified by an optimal cut-off and let T*^(*k*)^ be the corresponding set of statistics obtained from *k* permuted data sets. The null hypothesis of no difference between the two groups can be assessed by computing an estimate of the corresponding *p*-value (p*), obtained from the proportion of T*^(*k*)^ ≥ *T* [5,7]:

(1) $$p^{*}=\frac{\#\left(T^{*\left(k\right)}\geq T\right)}{k},$$

In the analysis of patient survival, *T* is usually a test statistic for the comparison of two survival curves or for the inference on the corresponding coefficient from a Cox regression model. For instance, a Cox model is usually employed to assess the association between a tumour marker evaluated at a specific cut-off and patient survival. The corresponding regression coefficient represents an estimate of the natural logarithm of the *HR* (ln(*HR*)) between the group of patients with marker values above the selected threshold and those with values below the threshold [2]. The estimate of ln(*HR*) is prone to an overestimation bias when the threshold is identified by a running procedure. Here, we propose a simple method to obtain an unbiased estimate of ln(*HR*) from the corresponding adjusted *p*-value (p*), which is estimated according to Equation (1).

Under the null hypothesis (H0: ln(*HR*) = 0), a normal asymptotic distribution for ln(*HR*) can be assumed [8]. Accordingly:

(2) $$\frac{{\left(\ln\left(HR\right)\right)}^{2}}{V}\overset{d}{\to }{\chi^{2}}_{1}$$

where *V* is the variance of ln(*HR*) and ${\chi^{2}}_{1}$ is a chi squared distribution with one degree of freedom (d.f.). The equation above is the basis for the application of the Wald test for the inference for the Cox regression coefficient [8].

Let p* be the adjusted *p*-value associated to ${\chi^{2}}_{1}$, estimated according to Equation (1), and let $\chi^{2*}$ be the quantile of the chi-squared distribution with 1 d.f. corresponding to p*. An estimate of the *HR* adjusted for the overestimation bias (HR*) is easily obtained by replacing ${\chi^{2}}_{1}$ with $\chi^{2*}$ in Equation (2) and solving for *HR*:

(3) $${\hat{HR}}^{*}=e^{sign\left(\ln\left(\hat{HR}\right)\right)\cdot \sqrt{\hat{V}\cdot \chi^{2*}}}$$

where the sign of $\ln\left(\hat{HR}\right)$ is introduced to preserve the direction of the association, i.e., to allow ${\hat{HR}}^{*}$ be <1 in the case of a positive association observed between the gene expression and the patient survival. Accordingly, a negative sign would be expected, for example, in the presence of a gene with a tumour suppression activity.

## 3. Statistical Validation of the Proposed Method to Control the Overestimation Bias: Analysing in Silico Data from Two Real Data Sets

### 3.1. The Data Sets

The statistical validation of the proposed method was performed using data from two large databases that were randomly split into a training set and a test set of equal sample size. The first data set, published by Cangelosi and collaborators, included information on 786 patients affected by NB, the most frequently occurring solid malignancy in infants, and 13,696 gene expression profiles [9]. Data were randomly split into a training set and a test set of equal sample size (*n* = 393). Their characteristics are summarized in Table 1. In the whole data set, there were 337 (43%) patients aged ≥ 18 months, *MYCN* amplification was observed in 153 (20%), and 373 (48%) were diagnosed in localised stages: namely, 143 in Stage 1, 125 in Stage 2, and 105 in Stage 3. There were 412 (53%) patients diagnosed with metastatic disease, including 320 at Stage 4 and 92 at Stage 4S. The number of observed deaths was 229. Random splitting allowed for a quite balanced distribution of the variables in the training and in the test sets, except for a slightly higher proportion of patients with disseminated disease in the former (55.5% vs. 49.4%). This was due to a higher proportion of Stage 4 patients (43.3% vs. 38.2%).

The second data set, published by Cavalli et al., included the expression profiles of 18,479 genes in 763 samples of medulloblastoma (MB), the most common malignant central nervous system tumour in children [10]. Patients aged ≥ 14 years (*n* = 186) and without follow up information (*n* = 78) were excluded from the analyses. Characteristics of the 499 considered patients are presented in Table 2. In the whole data set, 109 patients were 0–3 years of age at diagnosis (21.8%), 320 were 4–10 years of age (64.1%), and 70 were 11–13 years of age (14.0%). The number of males was 323 (64.7%). Group 4 was the most common molecular subgroup (*n* = 241, 48.3%) followed by Group 3 (*n* = 106, 21.2%), WNT (8.0%), and SHH (*n* = 112, 22.4%). Classic histology was observed in 279 patients (55.9%), desmoplastic in 65 (13.0%), large cell/anaplastic in 57 (11.4%), and extensive nodularity in 14 (2.8%). Metastatic disease was observed in 148 patients (29.7%), and 139 patients died of MB (27.9%). Random splitting allowed for a quite balanced allocation of the patients’ characteristics except for the male gender, which was slightly more represented in the test set (70.3% vs. 59.2%).

Due to the heavy computational burden, a convenience sample of 1000 genes in each database (the first ones included) was used for the analysis, as described in the following paragraph.

### 3.2. The Validation Procedure

For each gene included in the validation analysis, overall survival was estimated in the training set by selecting the optimal cut-off using a running procedure and adjusting the *HR* estimate, which was obtained from the corresponding univariable Cox model, using the method described in Section 2 and 10,000 random permutations. In the presence of no observed deaths in either group, the *HR* was estimated applying Firth’s correction, which is based on a penalized likelihood function [11].

The overestimation bias was estimated by computing the difference between the unadjusted *HR*s from the training set and the corresponding *HR*s from the test set, expressed on a logarithmic scale. The reduction in the overestimation bias was estimated by the corresponding difference obtained by replacing the unadjusted *HR*s with the adjusted ones. The same procedure was also performed using the median value as a cut-off. The results of this latter analysis are expected to provide an internal referent as this approach is not prone to an overestimation bias. All analyses were performed by separating the genes into potentially over- and under-expressed categories (*HR* > 1 and *HR* ≤ 1, respectively) on the basis of the corresponding coefficient of the Cox regression model, evaluating the expression value of each gene in the entire data set.

Both the survival and permutation analyses were performed using the R programming language [12]. The R libraries survival and coxphf were used for the survival analysis. The R script, including the routines for the validation procedures, are available in the Supplementary Materials (files HRValidCangelosi.R and HRValidCavalli.R) with the corresponding data sets (files DataSetCangelosi_1040.RData and DataSetCavalli_1000.RData) and the table containing the related results (files StatisticalValidationResultsCangelosi.csv and StatisticalValidationResultsCavalli.csv). All the other analyses were carried out using the STATA for Windows statistical package (release 13.1, Stata Corporation, College Station, TX, USA).

### 3.3. Results of the Statistical Validation on Real Data Sets

**Figure 1:**
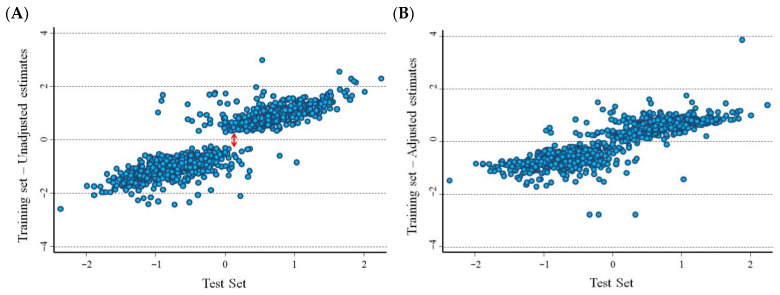
Association between the hazard ratio estimates, obtained from the training set and the test set, on a logarithmic scale. Data are from the Cangelosi et al. data set [9]. (**A**) Unadjusted values; (**B**) values adjusted for the overestimation bias. The double red arrow in panel A highlights the gap between unadjusted estimates in the training set around the null as a consequence of the overestimation bias. An outlier observed in the training set (−5.5) has been removed from panel B for aesthetic reasons.

Figure 1A,B show the association between the *HR* estimates, on a logarithmic scale, in the training set and in the test set of the Cangelosi et al. database [9]. Unadjusted estimates are displayed in Figure 1A, in which a gap is evident in the *HR* distribution around the null as a consequence of the overestimation bias. Such a gap was no longer evident after correction for the overestimation bias according to the procedure described in Section 2 (Figure 1B). The correlation between the estimates at the optimal cut-off in the training set and in the test set was very high (r = 0.904, *p* < 0.001 for unadjusted estimates, and r = 0.847, *p* < 0.001 for adjusted estimates, respectively). Similar figures were observed for the cut-offs based on median values (r = 0.928, *p* < 0.001, and r = 0.865, *p* < 0.001, for the unadjusted and adjusted estimates, respectively).

**Figure 2:**
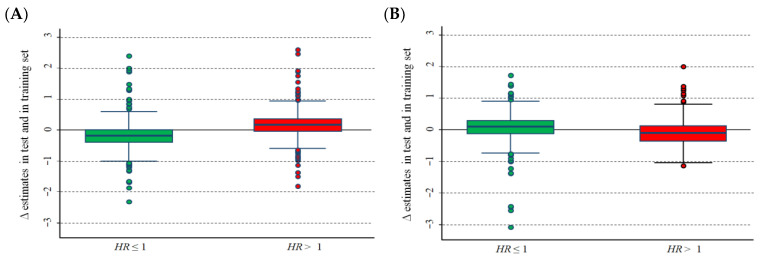

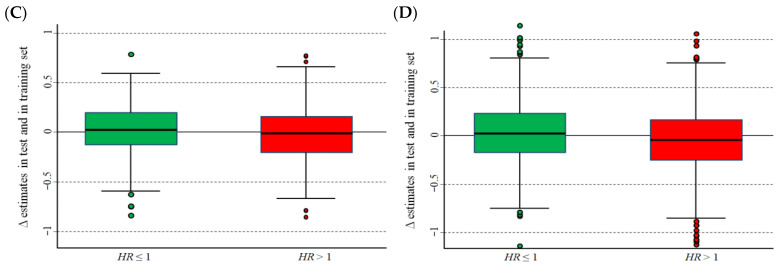
Difference (Δ) between the hazard ratio, on a logarithmic scale, estimated in the training set and the corresponding estimate in the test set. Data from the Cangelosi et al. data set [9]. (**A**) Optimal cut-off, unadjusted values; (**B**) optimal cut-off, values adjusted for the overestimation bias; (**C**) median cut-off, unadjusted values; and (**D**) median cut-off, values adjusted for the overestimation bias. Green boxes: *HR* ≤ 1; red boxes: *HR* > 1.

Figure 2A–D show the difference between the ln(*HR*) estimates calculated in the training set and in the test set of the Cangelosi et al. database [9]. As expected, the unadjusted estimates in the training set were, on average, lower than those obtained in the validation set for *HR* < 1 and higher for *HR* > 1 (Figure 2A). In more detail, the corresponding median values were −0.182 (interquartile range, IQR: −0.398–−0.012) and 0.173 (IQR: −0.036–0.364), respectively, corresponding to an estimation bias of −16.6% and 18.9%, respectively. Adjusted estimates (Figure 2B) provided values that slightly exceeded zero for *HR* < 1 (median value: 0.100; IQR: −0.118–0.297) and were slightly negative for *HR* > 1 (median value: −0.099; IQR: −0.359–0.122). Using the median value of each gene expression as a cut-off, the corresponding unadjusted estimates were: median value: 0.026; IQR: −0.126–0.195 for *HR* < 1 and median value: −0.012; IQR: −0.204–0.159 for *HR* > 1 (Figure 2C). The corresponding figures for the adjusted estimates were: median value: 0.022; IQR: −0.175–0.230 for *HR* < 1 and median value: −0.04; IQR: −0.251–0.165 for *HR* > 1, respectively (Figure 2D).

**Figure 3:**
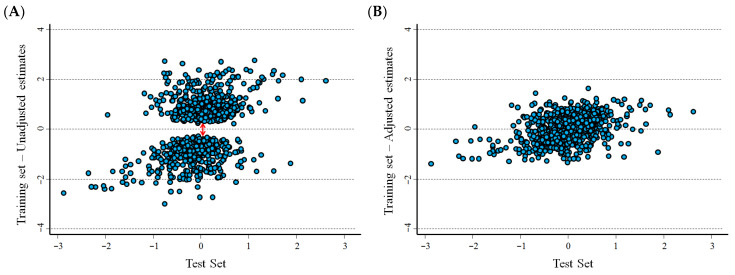
Association between the hazard ratio estimates, obtained from the training set and the test set, on a logarithmic scale. Data from the Cavalli et al. data set [10]. (**A**) Unadjusted values and (**B**) values adjusted for the overestimation bias. The double red arrow in panel A highlights the gap between unadjusted estimates in the training set around the null as a consequence of the overestimation bias.

Figure 3A,B show the association between estimates of the logarithm of the *HRs* in the training set and in the test set of the Cavalli et al. database [10]. In Figure 3A, a gap is evident between the distribution of the unadjusted *HR* around the null as a consequence of the overestimation bias. Such a gap disappeared after correction for the overestimation bias (Figure 3B). The correlation between the estimates at the optimal cut-off in the training set and in the test set was quite low (r = 0.402, *p* < 0.001, unadjusted estimates, and r = 0.399, *p* < 0.001, adjusted estimates). Similar estimates were observed for cut-offs based on median values (r = 0.465, *p* < 0.001, unadjusted estimates; and r = 0.446, *p* < 0.001, adjusted estimates).

**Figure 4:**
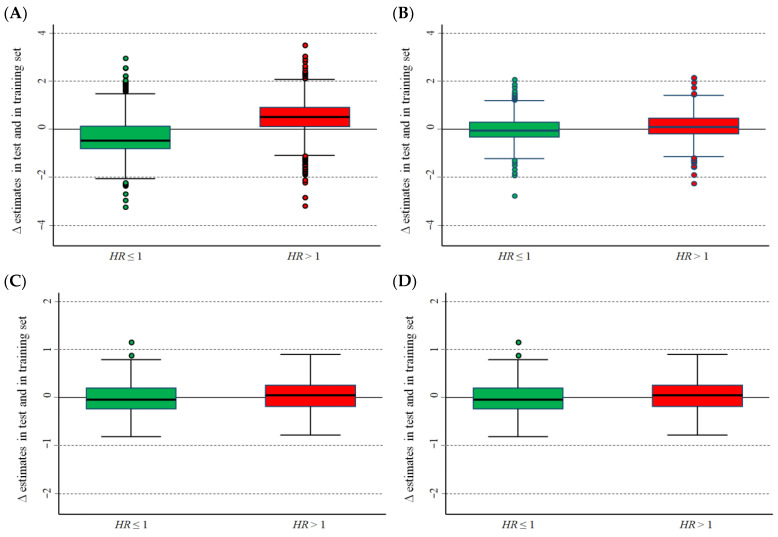
Difference (Δ) between the hazard ratio, on a logarithmic scale, estimated in the training set and the corresponding estimate in the test set. Data from the Cavalli et al. database [10]. (**A**) Optimal cut-off, unadjusted values; (**B**) optimal cut-off, values adjusted for the overestimation bias; (**C**) median cut-off, unadjusted values; and (**D**) median cut-off, values adjusted for the overestimation bias. Green boxes: *HR* ≤ 1; red boxes: *HR* > 1.

Figure 4A–D show the difference between the ln(*HR*) estimates calculated in the training set and in the test set. Median values of the optimal, unadjusted estimates in the training set were lower than those in the validation set for *HR* < 1 and higher for *HR* > 1 (Figure 4A). In more detail, the corresponding median values were −0.467 (interquartile range, IQR: −0.792–0.126) and 0.507 (IQR: 0.111–0.911), respectively, which corresponded to an estimation bias of −37.3% and 66.0%, respectively. The adjusted estimates at an optimal cut-off (Figure 2B) showed values close to zero for both *HR* ≤ 1 (median value: −0.064; IQR: −0.332–0.286) and for *HR* > 1 (median value: 0.092; IQR: −0.192–0.459). Using the median value as a cut-off, the corresponding estimates for unadjusted values were: median value: −0.051; IQR: −0.240–0.192 for *HR* <1 and median value: 0.043; IQR: −0.189–0.252 for *HR* > 1 (Figure 1C). For the adjusted values, the corresponding estimates were: median value: −0.051; IQR: −0.249–0.193 for *HR* < 1 and median value: 0.050; IQR: −0.221–0.216 for *HR* > 1 (Figure 1D).

## 4. Statistical Validation Using Simulated Sets of Randomly Generated Data

### 4.1. The Data Sets

A preliminary analysis under the null hypothesis of no association between gene expression profiles and patient survival was carried out using a simplified model. Five artificial data sets, each including 1000 randomly simulated expression values (corresponding to hypothetical “genes”), were randomly extracted from a normal distribution, arbitrarily setting the mean to four and the variance to one. The sample size varied from 20 to 100 (step 20), and a further sample of 200 statistical units (the “patients”) was also generated. Each sample was associated with a follow-up time randomly generated under the assumption of an exponential model for the survival probability, setting the event rate (lambda) to 0.1 arbitrary units. The follow-up time was right-truncated at 10 units to generate censored observations. The simulation process was entirely repeated after changing the lambda parameter to 0.3, thus obtaining, on the whole, 12,000 independent gene expression profiles. Adjusted ln(*HR*) estimates were obtained by 2000 random permutations for each simulated gene expression.

The overestimation bias was estimated by the average absolute difference between each ln(*HR*) estimate at the optimal cut-off and its expected value under the null, calculating the corresponding standard error (SE). The reduction of the overestimation bias by the proposed method was assessed by performing the same analysis on the adjusted ln(*HR*). The corresponding estimates, obtained using the median values as a cut-off, were also calculated to obtain a referent distribution. The overfitting related to the running method for the selection of an optimal cut-off was assessed, estimating the corresponding Type I error by the proportion of statistical significances of the likelihood ratio test associated with the *HR* estimates in the univariable Cox regression model. The usual nominal alpha level of 0.05 was assumed.

In order to obtain a preliminary estimate of the potential impact of sample size and event rates on the adjusted *HR* estimates, a set of simulated databases was also generated under the hypothesis of an association between gene expression level and patient survival. In each data set, two hidden balanced groups of patients were assumed, each associated with a different constant event rate (namely, lambda = 0.1 for the first group and lambda = 0.3 for the second group; arbitrary units). Gene expression levels for the two hidden groups were generated, assuming a normal distribution with mean = 0 for the first group and mean = 1 for the second group and setting both variances to 1. Thus, two partially overlapped distributions were obtained. All the analyses were repeated and a mean = 5.0 was set for the second group, corresponding to a nearly completely separated distribution. Assuming such a binormal model for the gene expression distribution allowed us to easily obtain both the value of the best expected cut-off, which corresponded to the average between the means of the two hidden classes (optimal discriminant threshold, ODT), and the corresponding “true” *HR* expected at the optimal cut-off according to the following equation, the demonstration for which is reported in the Supplemental Materials (Supplemental Figure S1, file DemonstrationEquation4.pdf):

(4) $$HR_{opt}=\frac{\lambda_{0}+\lambda_{1}}{\lambda_{0}\Phi \left(\frac{ODT-\mu_{0}}{\sigma_{0}}\right)+\lambda_{1}\Phi \left(\frac{ODT-\mu_{1}}{\sigma_{1}}\right)}-1$$

where *λ*_0_ and *λ*_1_ represent the event rates in the two hidden groups, respectively, *µ*_0_ and *µ*_1_ the means of the two corresponding distributions, respectively, *σ*_0_ and *σ*_1_ the corresponding standard deviations, respectively, and Φ is the standard normal cumulative distribution function.

Non-valid estimates of the adjusted *HR* were identified in each analysis. These corresponded to values more biased than the unadjusted ones (i.e., higher values of adjusted estimates when the unadjusted *HR* was higher than 1, and lower values of adjusted estimates when the unadjusted *HR* was lower than 1). Outliers were defined as *HR*s estimated at the optimal cut-off more than three times higher than the expected value calculated according to Equation (4). The overestimation bias and its reduction by the proposed method were estimated by comparing the adjusted and unadjusted *HR* estimates with the corresponding expected *HR*. Finally, a statistical power at the alpha nominal level of 0.05 was also estimated.

The sample size was allowed to vary from 20 to 160, and the number of simulated gene expressions was set to 2000 for each data set. The adjusted *p*-values for the *HR* at the optimal cut-off were obtained by 5000 random permutations.

All simulation analyses were carried out by ad hoc developed scripts in the R programming language [12] (files SimulAdjHRH0.R and SimulAdjRH1.R, Supplemental Materials), and analysed by the STATA for Windows statistical package (release 13.1, Stata Corporation, College Station, TX, USA).

### 4.2. Results of Analysis of Simulated Data Sets under the Null Hypothesis

**Figure 5:**
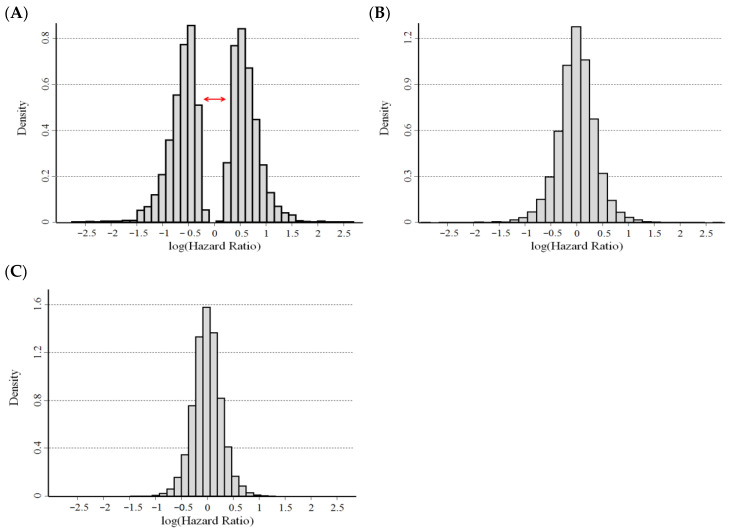
Histograms of the distribution of the hazard ratio estimates, on a logarithmic scale, obtained at an optimal cut-off on a simulated data set of 12,000 gene expressions under the null hypothesis of no association between survival and expression levels. (**A**) Unadjusted values and (**B**) values adjusted for the overestimation bias. (**C**) Values obtained using the median as a cut-off. The double red arrow in panel A highlights the gap between unadjusted estimates around the null as a consequence of the overestimation bias.

Figure 5 shows the distribution of the ln(*HR*) estimates for the pool of the 12,000 simulated gene expressions, obtained: (a) at an optimal cut-off, without adjustment for the overestimation bias (panel A); (b) at an optimal cut-off, adjusting for the overestimation bias (panel B); and (c) using the median value as an a priori selected cut-off (panel C). The unadjusted optimal ln(*HR*) is clustered into two groups above and below the expected value under the null (zero) as a consequence of the overestimation bias (Figure 5A). The adjustment using the proposed method strongly reduced the bias (Figure 5B), producing a histogram that was very similar to the histogram obtained using the median expression level as a cut-off (Figure 5C). However, this latter figure shows a slightly smaller variance, indicating that some residual bias remained after the adjustment procedure.

The results of the analysis by event rate and sample size are reported in Table 3. The Type I error associated with unadjusted *HR* estimates was very large, indicating a strong overfitting (0.371 and 0.376 for the pool of the data for lambda = 0.1 and lambda = 0.3, respectively). Such a bias was positively associated with the sample size and seemed to be unrelated to the event rate. Estimates adjusted by permutation analysis produced values very close to the nominal alpha level (0.05) for both the pool of samples and for each considered sample size, indicating a very good control of the overfitting. Such estimates were consistent with those obtained using the median value as a cut-off. An analysis of the corresponding standard errors, reported in the right part of Table 3, indicated a strong reduction of the overestimation bias by the newly proposed method. For instance, for the pool of the data, the SE was 0.777 for the unadjusted estimates and 0.489 for the adjusted estimates for lambda = 0.1, and 0.644 and 0.374, respectively, for lambda = 0.3. An analysis by the number of subjects in each sample indicated, as expected, an inverse association between the SE and the sample size for both adjusted and unadjusted estimates. The corresponding SE estimates, obtained using the median value as cut-off, were lower than those for the adjusted estimates for each considered group, indicating the presence of some residual bias, which seemed positevely associated with the sample size and inversely associated with the event rate.

### 4.3. Results of Analysis of Simulated Data Sets under the Alternative Hypothesis

Supplemental Table S2 shows the proportion of non-valid estimates of the adjusted *HR* by sample and the proportion of the observed outliers. When the difference between the two hidden subgroups was small (mean difference Δ*µ* = 1 arbitrary unit, corresponding to a true *HR* at the optimal cut-off of 1.47), the proportion of the non-valid, adjusted *HR* was low in all considered groups. A higher proportion (8.2%) was observed for a very small sample size (*n* = 20), corresponding to a lower overlap between the two hidden distributions (Δ*µ* = 5, corresponding to a true *HR* of 2.95). For a higher sample size, the proportion was very small and rapidly decreased with rising the number of samples. The occurrence of outliers was higher in the first group (*n* = 20, 7.7% for Δ*µ* = 1 and 10.1% for Δ*µ* = 5, respectively) and decreased with the increasing sample size, more rapidly in correspondence with the highest separation between the two hidden classes (Δ*µ* = 5).

Supplemental Table S3 shows the comparison between the adjusted and unadjusted *HR* estimates by sample size. A strong reduction in the overestimation bias was evident in all analyses. Corresponding to a small difference between the two hidden classes (Δ*µ* = 1), the adjusted *HR* estimates remained slightly higher than the corresponding expected true values. This indicated a small residual bias which did not seem related to the sample size. In the presence of a higher separation between the two hidden classes (Δ*µ* = 5), the correction for overfitting was excellent for *n* < 80 and quite good for *n* = 80 but tended to be overconservative for a higher number of samples, producing estimates lower than the expected (2.24 vs. 2.95 for 120 samples and 2.02 vs. 2.95 for 160 samples, respectively).

The statistical power of the permutation test for the inference for the *HR* estimates is reported in Supplementary Table S4, which also shows the corresponding estimates for the unadjusted HR values. The power of the permutation test was very low in the presence of a small separation between the two hidden classes (Δ*µ* = 1), ranging from 11.0% for *n* = 20 to 58.5% for *n* = 160. In correspondence with a smaller overlap between the two classes (Δ*µ* = 5), the statistical power was still low for *n* = 20 (45.8%) and rapidly increased with an increase in the sample size (from 73.8% for *n* = 40 to 100% for *n* = 160).

## 5. Application to a Real Data Set for the Evaluation of Potential Prognostic Markers in Patients with Stage 4S Neuroblastoma

### 5.1. The Data Set

Stage 4S NB (S stands for “special”) is a metastatic disease occurring in the first year of life. It is characterized by metastasis that is limited to liver, skin, or bone marrow, with an infiltration of less than 10% of [13,14]. In general, Stage 4S NB has a good prognosis and a high rate of spontaneous regression. However, 10–20% of cases are still destined to demonstrate disease progression and eventual death [13,14].

In recent years, many genes have been investigated in relation to NB progression, including Stage 4S disease [14,15]. For instance, Parodi et al. [6] analysed the association between the expression of three genes belonging to the E2F family (namely, *E2F1*, *E2F2*, and *E2F3*) and the event-free survival (EFS) of 134 Stage 4S NB patients. Data were drawn from three publicly available databases, stored in the on-line data bank R2 Genomics Analysis and Visualization Platform (http://r2.amc.nl, accessed on 17 December 2019): namely, Kocak-649, Oberthuer-251, and SEQC-RPM [16,17,18]. For each data set, patients were split into two groups of approximately equal size using the median value of each gene expression considered. The related *HR* estimates were calculated using the Cox regression model, applying the Firth correction in the case of no events in either group [11]. For each gene, a common meta-analysis estimate of the *HR* (*mHR*) across the three databases considered was obtained by the random effect model proposed by DerSimonian and Laird [19]. Previously published results identified an association between EFS and high expression levels of *E2F3* (*mHR* = 3.9, 95%CI: 1.7–9.1), but not of *E2F1* (*mHR* = 1.6, 95%CI: 0.80–3.3) or *E2F2* (*mHR* = 1.4, 95%CI: 0.70–2.9) [6]).

In this investigation, we performed a reanalysis of these data, splitting the patients on the basis of an optimal cut-off identified using a running analysis instead of the median value, as described above, to show the utility of the proposed method in practice. An estimate of the *HR,* adjusted for the overestimation bias, was produced according to Equation (3), and the corresponding *mHR* was obtained by applying the method of DerSimonian and Laird [19]. Original cut-offs corresponding to the median value of each gene expression, the related interquartile range (IQR), and the newly selected optimal cut-offs are reported in Table 4. The data sets and the R script used for the analyses are available in the Supplementary Materials (files NB4S.csv and SurvNB4S.R, respectively).

### 5.2. Results of the Application of the Proposed Method

**Figure 6:**
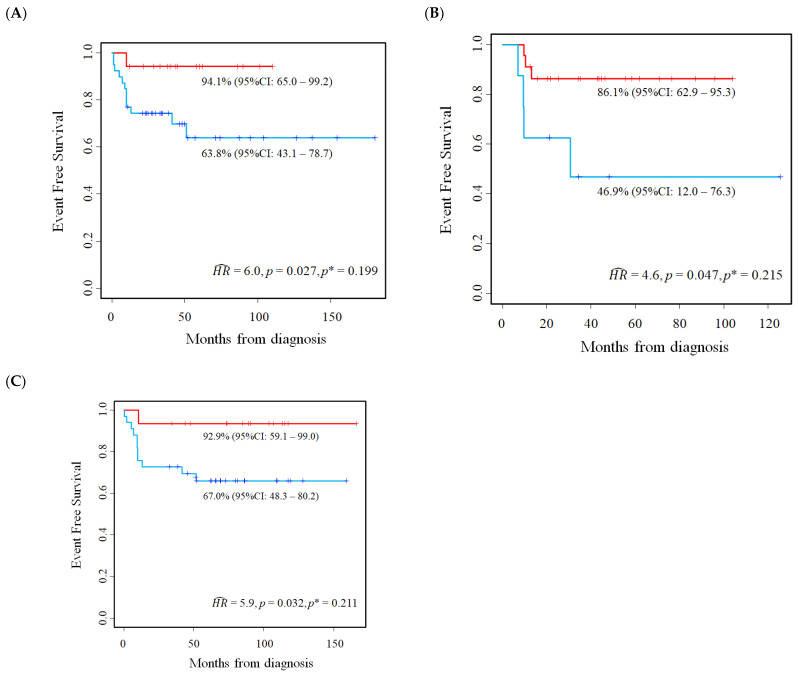
Kaplan–Meier event-free survival curves for the evaluation of *E2F1* gene expression as a prognostic marker in Stage 4S neuroblastoma patients at the optimal cut-off. Data extracted from three public databases: (**A**) Kocak; (**B**) Oberthuer; and (**C**) SEQC. p* = *p*-value adjusted for the overestimation bias by permutation analysis. Five-year EFS estimates and the related 95% confidence intervals are shown.

The results of the analysis of the association between *E2F1* expression, evaluated at the optimal cut-off, and the EFS of the patients in the three studied cohorts are presented in Table 5. The corresponding Kaplan–Meier survival curves are displayed in Figure 6A–C. An association between *E2F1* expression and EFS was found using the optimal cut-off as a threshold (unadjusted *mHR* = 5.3, 95%CI: 1.9–15.0). After the adjustment of the overestimation bias, the association was reduced but remained statistically significant, with an adjusted *mHR* = 3.1 (95%CI: 1.1–8.9) that was approximately twice that obtained by the previous analysis, which was based on the median value of the gene expression (*mHR* = 1.6, Table 5).

**Figure 7:**
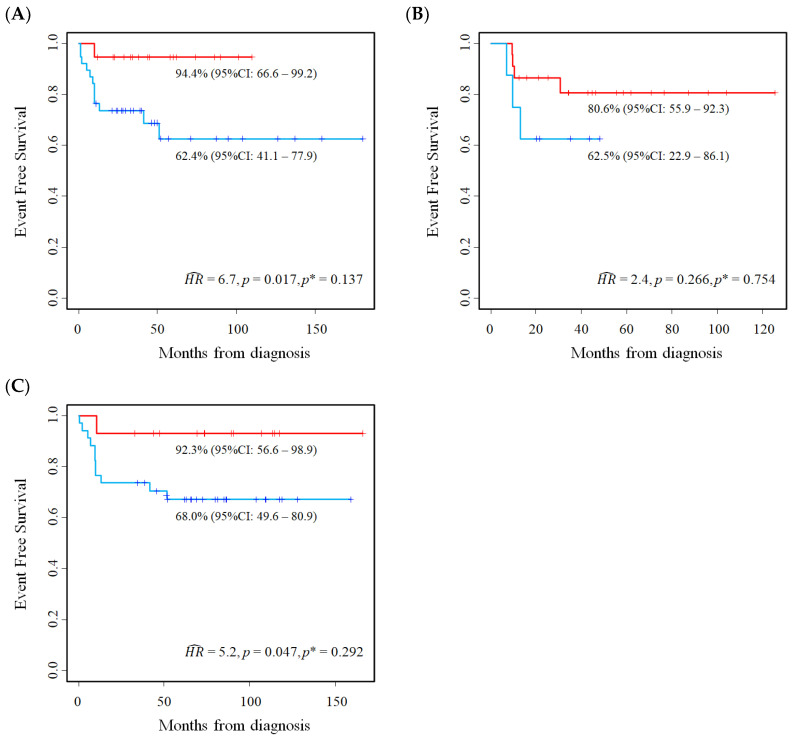
Kaplan–Meier event-free survival curves for the evaluation of *E2F2* gene expression as a prognostic marker in Stage 4S neuroblastoma patients at the optimal cut-off. Data extracted from three public databases: (**A**) Kocak; (**B**) Oberthuer; and (**C**) SEQC. p* = *p*-value adjusted for the overestimation bias. Five-year EFS estimates and the related 95% confidence intervals are shown.

Table 6 shows the estimates of the association between *E2F2* expression and EFS, while the corresponding Kaplan–Meier survival curves are displayed in Figure 7A–C. Higher levels of gene expression were associated with a poorer EFS (unadjusted *mHR* = 3.9, 95%CI: 1.4–10.9). However, after correcting for the overestimation bias the association was reduced and no longer significant (adjusted *mHR* = 2.2, 95%CI: 0.79–6.3).

**Figure 8:**
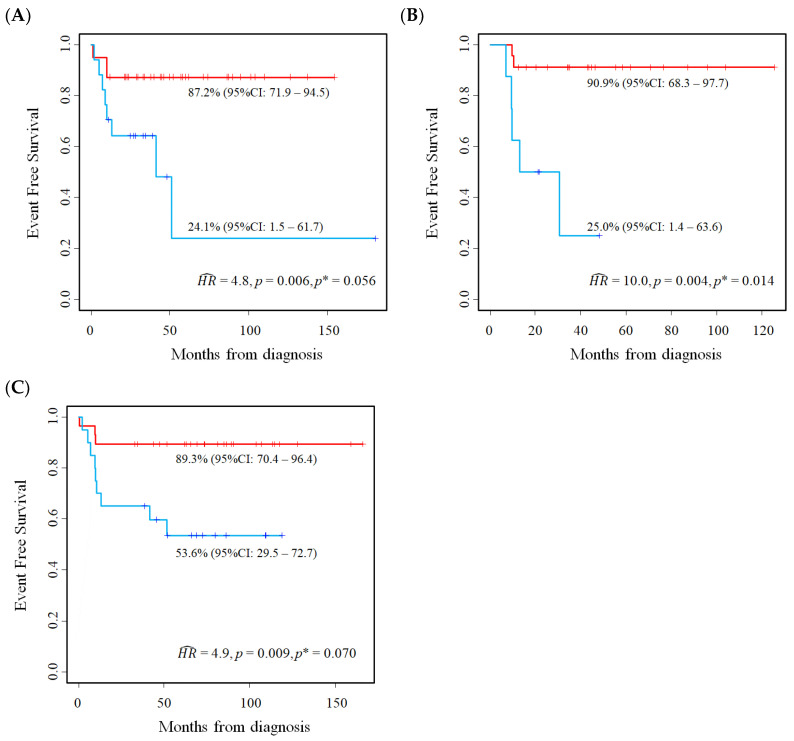
Kaplan–Meier event-free survival curves for the evaluation of *E2F3* gene expression as a prognostic marker in Stage 4S neuroblastoma patients at the optimal cut-off. Data extracted from three public databases: (**A**) Kocak; (**B**) Oberthuer; and (**C**) SEQC. p* = *p*-value adjusted for the overestimation bias. Five-year EFS estimates and the related 95% confidence intervals are shown.

Table 7 shows the association between *E2F3* expression and EFS in the three studied cohorts. The related Kaplan–Meier survival curves are displayed in Figure 8A–C. Higher values of *E2F3* were associated with a poorer outcome both in the original analysis, based on the median value cut-off, and in the new analyses, based on the optimal cut-off. In particular, the meta-analysis estimates of association were similar after adjusting the overestimation bias (original *mHR* = 3.9, 95%CI: 1.7–9.1; adjusted *mHR* at the optimal cut-off: 3.8, 95%CI: 1.8–8.2).

## 6. Validation on an Independent Cohort of the Results Described in the Previous Section

### 6.1. Patients and Methods

An immunofluorescence assay was performed to evaluate the expression level of the transcription factor E2F1 using formalin-fixed, paraffin-embedded, NB primary samples (4-μm thick) from an independent cohort of 38 patients with Stage 4S NB.

Patients belonged to a multi-institution, retrospective series of primary NB tissue sections with Stage 4S disease, diagnosed between December 2000 and October 2011 in 27 centres of the Italian Association of Paediatric Haematology and Oncology (AIEOP) [14]. The tumour tissue specimens were stored in the BIT-Gaslini Biobank of the IRCCS Istituto Giannina Gaslini, Genova, Italy. The patient data were downloaded from the Italian Neuroblastoma Registry (INBR) of AIEOP. The clinical characteristics of NB patients were collected in pseudo-anonymized manner and stored in a secure system in a database located at the Italian Inter-University Consortium CINECA headquarters in Italy, which received the ISO 9001:2015 Quality Management System certification and the ISO/IEC 27,001:2013 Information Security Management System certification. Medical records were abstracted at each institution, and clinical data, including age at diagnosis, sex, stage, *MYCN* status, DNA index, histology, and outcome, were collected. The patients were staged according to the International Neuroblastoma Staging System [20]. 

The studied cohort included 38 patients aged <12 months; 34 of them had a normal *MYCN* status and all were treated in accordance with the therapeutic guidelines of an ad hoc SIOPEN (International Society of Paediatric Oncology Europe Neuroblastoma) protocol [21]. Fourteen of them experienced tumour relapse (1 local and 13 metastatic) (Supplemental Table S1).

An immunofluorescence assay was carried out as previously described [22] using the mouse monoclonal antibody E2F-1 (KH95, sc-251) (Santa Cruz Biotechnology, Dallas, TX, USA) and isotype-matched, non-binding mAbs in all antibody staining experiments to avoid nonspecific reactivity. Counterstaining of the nuclei was performed with DAPI (4′,6-diamidino-2-phenylindole) (Vector Laboratories, Peterborough, UK). The results were photographically documented using fluorescence microscope Axio Imager M2 equipped with ApoTome System (Carl Zeiss, Oberkochen, Germany). Each tumour area tested by immunofluorescence contained more than 60% NB cells, as assessed by histological examination. The evaluation of immunofluorescence-positive tumour cells was performed on serial tissue sections, thus allowing quantification in tumour areas selected by the pathologist. The proportion of immunofluorescence-positive cells counted was at least 100–1000 cells and was reported as percentage for the subsequent statistical analysis.

### 6.2. E2F1 Protein Expression in Primary Neuroblastoma Tissue Samples

**Figure 9:**
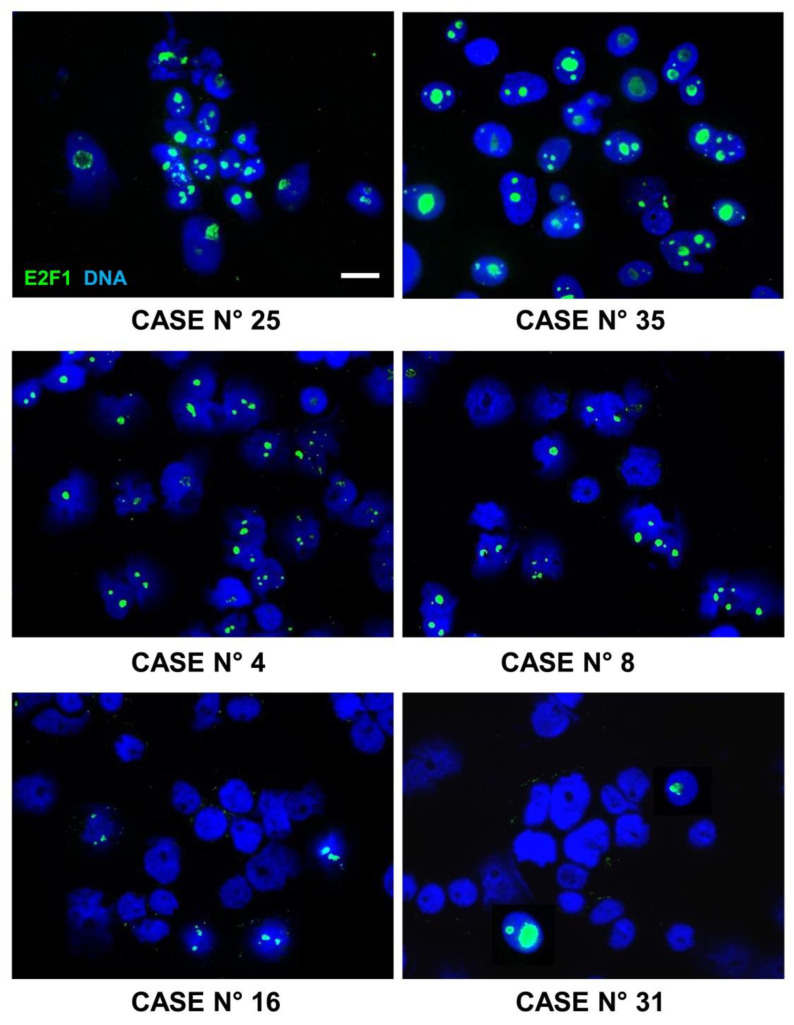
Expression of E2F1 protein in Stage 4S primary NB tissues. Immunofluorescence assay was performed using the anti-E2F1 antibody (green). Images are representative of various percentages of brilliant green nuclear staining for E2F1 on six tissues among the 38 patients examined, displaying high (cases 25 and 35), medium (cases 4 and 8), and low (cases 16 and 31) numbers of positive nuclei. Cells were counterstained with DAPI to visualize nuclei (blue). Three independent experiments were performed. Scale bar: 10 µm.

**Figure 10:**
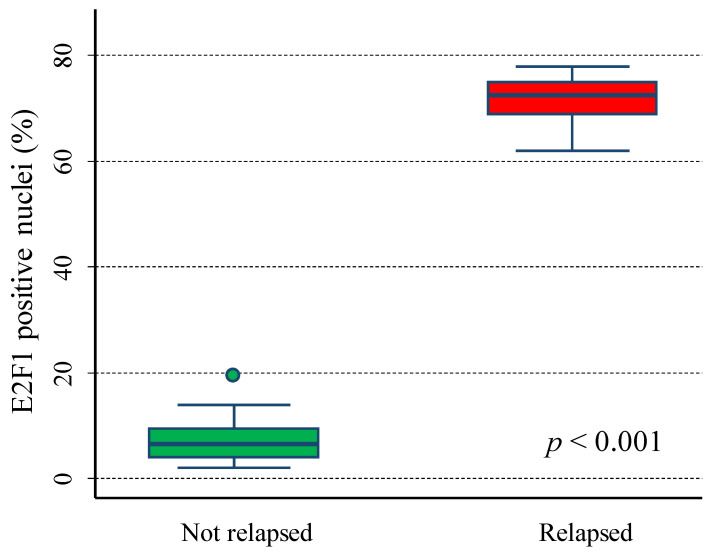
Relative *E2F1* expression levels in not relapsed and relapsed patients. Green box: not relapsed patients; red box: relapsed patients.

The analysis highlighted various percentages of brilliant green nuclear staining for E2F1 in the 38 analysed specimens (Figure 9 and Supplemental Table S1). The percentage of positive nuclei ranged from 2% to 78%. All relapsed patients showed many E2F1-positive nuclei in their tumour tissues, while NB in complete remission expressed a lower amount of E2F1 positive nuclei (*p* < 0.001, Mann–Whitney U test). In more detail, the median value was 6.55 among the not-relapsed patients (IQR: 4.0–9.5) and 67.5 among the relapsed ones (IQR: 69.0–75.0, Figure 10).

## 7. Discussion

Running tests are frequently applied in early studies to evaluate the association between potential tumour markers and patient survival. The overestimation bias, i.e., the production of an “overoptimistic” estimate of association, is a well-known limitation of such investigations. Many algorithms are available for obtaining adjusted *p*-values to test the hypothesis of no association (e.g., H0: *HR* = 1), ranging from the very conservative Bonferroni’s correction to resampling procedures that include permutation analysis [5,7]. Exploiting the log-normal asymptotic distribution of the *HR* under the null hypothesis, we developed a very simple, test-based method to remove the overestimation bias, beginning with an adjusted estimate of the corresponding *p*-value obtained from a set of random permutations [23]. A statistical validation of our method, using both real and simulated data, indicated that the overestimation bias was largely removed from the *HR* estimates. However, simulated data revealed some residual overestimation bias under the null that was larger in correspondence to either a lower event rate or a smaller sample size. Estimates of the associated alpha value indicated that such a residual bias was not associated to an inflation of the type I error, which, conversely, was very high for the unadjusted *HR* estimates. On the contrary, the bias correction under H1, when estimates far from the null are more likely to be observed, could provide an over-conservative adjustment; this conclusion emerged from the analyses of both real and simulated data set. For instance, the shape of the plot in Figure 1B, obtained from the analysis of the Cangelosi et al. [9] data set, seems consistent with this hypothesis in that it shows a “shrinkage” in the adjusted values at the extreme of the ln(*HR*) distribution. A similar pattern was also observed when analysing the Cavalli et al. data set [10] (Figure 3B), even if the picture was affected by a high variability, as is also indicated by the low correlation between estimates from the training set and the test set. Interestingly, a strong reduction in the overestimation bias by the new, proposed method was clearly also observed in the analysis of the Cavalli et al. [10] database. Unfortunately, the heavy computational burden of the running analysis prevented us from performing a more complete statistical validation. Further investigations could provide more precise estimates that would be useful for refining the proposed method, evaluating the effect of different sample size, marker distributions, association between marker values and survival probability, different survival functions, and the distribution of the time censoring.

Our method is not intended to replace validation procedures that remain the gold standard for assessing the generalizability of observed results. On the contrary, it should be applied when neither an external cohort is available, or when internal validation is unreliable due to an insufficient sample size. In the latter situation, the application of standard methods of internal validation for classification tasks, such as the *k*-fold cross-validation or the leave-one-out cross-validation, have sometimes been applied. However, they can be prone to confounding bias in a survival analysis since they can only be performed ignoring the follow-up time and limiting the permutation procedure to the outcome variable (dead/alive, relapsed/not relapsed, etc.). Sometimes they represent a quite forced choice when information on the follow-up time is not available, a situation often occurring in the retrospective analysis of published data. Nevertheless, these approaches should provide reliable results only in the absence of censoring, i.e., in the analysis of closed cohorts [24], or when the outcome of interest is not dependant on the length of the follow-up (for example, in the evaluation of the response of cancer patients to some therapeutic approach) [4].

In order to illustrate our method in an actual framework, we chose the data set previously included in the study by Parodi et al. [6]. Analysing the data using a standard procedure, they provided some ambiguous results about the potential prognostic role of *E2F1* gene expression in patients with Stage 4S NB. For instance, *HR*s estimates from cut-offs selected a priori could not clearly assess the association between a patient’s survival and the gene expression, while a mere running approach, without adjustment for the overfitting, would have provided a biased, meta-analytic estimate of the corresponding *HR*. Our proposed method, coupled with the standard meta-analysis technique by DerSimonian and Laird [19], allowed for the identification of *E2F1* as a new gene with potential oncogenic activity in NB 4S, though it had escaped previous standard methods of analysis in the original study [6]. Using our new proposed method, we verified even the previous finding of an inverse association between *E2F3* expression and NB patient survival. Both results were confirmed by biological validation using immunofluorescence analysis, in the present study with regard to the expression of *E2F1*, and in the original investigation for *E2F3* [6]. *E2F1* and *E2F3* are members of the E2F family, which plays an important role in regulating gene transcription, cell cycle, proliferation, and apoptosis [25]. Interestingly, the role of *E2F1* in NB prognosis has recently been demonstrated in a large investigation by Wang et al. [26], who reported an association between the gene expression and both *MYCN* amplification and a higher age at diagnosis, two major indicators of poor prognosis in NB patients. The authors also reported that *E2F1* and *E2F3* shared similar downstream transcriptional features, and that the high expression of both genes was significantly enriched in the cell cycle signalling pathway [26]. Even if these findings support our observations, it should be noted that Stage 4S NB is a malignancy with very peculiar characteristics, including the tendency to regress spontaneously [14]. Accordingly, the potential prognostic role of *E2F1* in Stage 4S patients, highlighted by the results of this study, needs to be validated in independent cohorts.

A limitation of our method is the absence of a control for potential confounders, such as the clinical and demographic characteristics of the patients. A confounding bias was unlikely in the reanalysis of the dataset of patients affected by Stage 4S neuroblastoma because the analysed patients were quite homogeneous for the major known prognostic factors, including age at diagnosis, stage, and amplification of the *MYCN* proto-oncogene. In a more general framework, in the early stage of selection of new potential markers for diagnostic or prognostic purposes, only simple, univariable analyses are usually carried out [27,28]. However, further studies aimed at applying the proposed method in the presence of external variables could be desirable. For instance, residual models represent a simple procedure to obtain marker distributions, including gene expression profiles, adjusted by the effects of one or more potential confounders [29]. Additional studies could evaluate the application of our method to the confounding-adjusted distribution of newly proposed tumour markers. Another limitation of the proposed method is that it is applied to binary splitting only. Stratification into two groups is the most natural choice in clinical framework, where most decisions are naturally binary (e.g., treatment or no treatment) [1]. However, in some instances, a classification based on three or, less frequently, more than three cut-offs is advisable. The possibility to extend the proposed method to multi-class analyses could then be explored. Finally, our method was applied to two real, in silico data sets, ignoring the assumptions of the Cox regression model that provided the *HR* estimates [2]—in particular, the proportional hazard assumption. However, even if severe violations of this assumption occur, the *HR* remains an estimator of an average relative risk across the whole follow-up time. Such an estimate can be useless or hardly interpretable from a clinical point of view, but it would not invalidate the statistical validity of the estimates obtained by our approach.

In conclusion, the results of our study, even if they are partially still preliminary, indicate that the proposed simple, test-based method is a new, useful tool for supporting the identification of new potential tumour markers for prognostic purposes.

## 8. Conclusions

In this paper we describe a simple, test-based method to control the overestimation bias in the estimate of the *HR* by a running procedure. A preliminary statistical validation indicated that our method is able to remove such a bias and, accordingly, it could be useful for many applications in oncology studies.

Combining the method with a standard meta-analysis approach allowed for the identification of *E2F1* as a new, potential oncogene for paediatric patients affected by Stage 4S NB. This finding was confirmed by an immunofluorescence analysis.

Further studies could extend our method to clinical estimators in oncology other than the *HR* and to different study designs.

## Figures and Tables

**Table 1:** Main characteristics of the data sets selected from the validation analyses from the Cangelosi et al. database [9].

	Whole Data (*n* = 786)	Training Set (*n* = 393)	Test Set (*n* = 393)
Patient Characteristics	N (%)	N (%)	N (%)
Age at diagnosis			
<18 months	449 (57.1)	228 (58.0)	221 (56.2)
≥18 months	337 (42.9)	165 (42.0)	172 (43.8)
*MYCN* status			
Not amplified	629 (80.0)	312 (79.4)	317 (80.7)
Amplified	153 (19.5)	78 (19.8)	75 (19.1)
Missing	4 (0.5)	3 (0.8)	1 (0.2)
Disease extension			
Localised	373 (47.5)	174 (44.3)	199 (50.6)
Disseminated	412 (52.4)	218 (55.5)	194 (49.4)
Missing	1 (0.1)	1 (0.2)	0 (0.0)
INSS Stage			
1	143 (18.2)	56 (14.2)	87 (22.1)
2	125 (15.9)	67 (17.0)	58 (14.8)
3	105 (13.4)	51 (13.0)	54 (13.7)
4	320 (40.7)	170 (43.3)	150 (38.2)
4s	92 (11.7)	48 (12.2)	44 (11.2)
Missing	1 (0.1)	1 (0.3)	0 (0.0)
Deaths	229 (29.1)	116 (29.5)	113 (28.8)

**Table 2:** Main characteristics of the data sets selected from the validation analyses from the Cavalli et al. database [10].

	Whole Data (*n* = 499)	Training Set (*n* = 250)	Test Set (*n* = 249)
Patient Characteristics	N (%)	N (%)	N (%)
Age at diagnosis			
0–3 years	109 (21.8)	57 (22.8)	52 (20.9)
4–10 years	320 (64.1)	163 (65.2)	157 (63.0)
11–13 years	70 (14.0)	30 (12.0)	40 (16.1)
Gender			
Males	323 (64.7)	148 (59.2)	175 (70.3)
Females	173 (34.7)	99 (39.6)	74 (29.7)
Missing	3 (0.6)	3 (1.2)	0 (0.0)
Molecular subgroups			
WNT	40 (8.0)	27 (10.8)	13 (5.2)
SHH	112 (22.4)	58 (23.2)	54 (21.7)
Group 3	106 (21.2)	54 (21.6)	52 (20.8)
Group 4	241 (48.3)	111 (44.4)	130 (52.2)
Histology			
Classic	279 (55.9)	137 (54.8)	142 (57.0)
Desmoplastic	65 (13.0)	30 (12.0)	35 (14.1)
Large cell/anaplastic	57 (11.4)	27 (10.8)	30 (12.0)
Extensive nodularity	14 (2.8)	7 (2.8)	7 (2.8)
Not available	84 (16.8)	49 (19.6)	35 (14.1)
Disease extension			
Localised	304 (60.9)	149 (59.6)	155 (62.2)
Disseminated	148 (29.7)	78 (31.2)	70 (28.1)
Missing	47 (9.4)	23 (9.2)	24 (9.6)
Deaths	139 (27.9)	65 (26.0)	74 (29.7)

**Table 3:** Results of the analysis of 12,000 simulated gene expression profiles under the null hypothesis of no association between gene expression and patient survival.

	Type I Error *			Standard Error of ln(*HR*)		
Sample Size	Unadjusted *HR* Estimates	Adjusted *HR* Estimates	*HR* Estimates on Median Value	Unadjusted *HR* Estimates	Adjusted *HR* Estimates	*HR* Estimates on Median Value
Event rate = 0.1 u^−1^						
20	0.127	0.048	0.042	0.842	0.659	0.591
40	0.311	0.044	0.037	0.849	0.520	0.394
60	0.358	0.055	0.056	0.795	0.463	0.328
80	0.408	0.049	0.046	0.754	0.432	0.279
100	0.459	0.049	0.047	0.722	0.397	0.255
200	0.564	0.056	0.052	0.688	0.411	0.188
Pooled	0.371	0.050	0.047	0.777	0.489	0.363
Event rate = 0.3 u^−1^						
20	0.136	0.046	0.058	0.747	0.564	0.537
40	0.303	0.056	0.052	0.704	0.404	0.347
60	0.388	0.046	0.053	0.651	0.338	0.277
80	0.436	0.051	0.045	0.613	0.312	0.237
100	0.436	0.047	0.044	0.583	0.278	0.203
200	0.554	0.062	0.055	0.544	0.261	0.147
Pooled	0.376	0.051	0.051	0.644	0.374	0.317

U = arbitrary time units. * Estimated at the nominal alpha value of 0.05.

**Table 4:** Median, inter-quartile range (IQR) and optimal cut-off of the studied markers in each data set.

Marker	Data Set	Median	IQR	Optimal Cut-Off
*E2F1*	Kocak	10.2	9.3–11.3	9.5
	Oberthuer	−0.250	−0.354–0.079	−0.026
	SEQC	4.8	4.0–5.6	4.092
*E2F2*	Kocak	11.7	10.8–12.7	11.0
	Oberthuer	−0.010	−0.048–0.109	0.099
	SEQC	4.2	3.5–4.8	3.5
*E2F3*	Kocak	11.5	10.9–12.1	11.8
	Oberthuer	−0.132	−0.347–0.079	0.035
	SEQC	4.0	3.6–4.5	4.1

**Table 5:** Hazard ratios for the association between event-free survival of patients with Stage 4S neuroblastoma and *E2F1* gene expression, categorized on the basis of the median expression value and on the optimal cut-off.

		Cut-Off on Median Value		Optimal Cut-Off		Optimal Cut-Off (Adjusted) *	
Databases	N/E	*HR*	95%CI	*HR*	95%CI	*HR*	95%CI
Kocak	56/13	1.9	0.61–5.7	6.0	0.79–46.5	3.8	0.50–29.3
Oberthuer	30/7	1.3	0.30–6.0	4.6	1.0–20.8	2.6	0.58–11.6
SEQC	48/12	1.6	0.50–5.0	5.9	0.76–45.4	3.7	0.48–28.7
Combined	134/32	1.6	0.80–3.3	5.3	1.9–15.0	3.1	1.1–8.9

N/E = amount of samples/events; *HR* = hazard ratio; 95%CI = 95% confidence interval; combined = combined estimates by the DerSimonian and Laird meta-analysis method. * Estimates adjusted by the overfitting bias, according to Equation (3).

**Table 6:** Hazard ratios for the association between event-free survival of patients with Stage 4S neuroblastoma and *E2F2* gene expression, categorized on the basis of the median expression value and on the optimal cut-off.

		Cut-Off on Median Value		Optimal Cut-Off		Optimal Cut-Off (Adj) *	
Databases	N/E	*HR*	95%CI	*HR*	95%CI	*HR*	95%CI
Kocak	56/13	2.0	0.64–6.0	6.7	0.87–51.8	4.5	0.61–36.2
Oberthuer	30/7	1.4	0.31–6.1	2.4	0.54–10.9	1.3	0.28–5.7
SEQC	48/12	1.0	0.34–3.2	5.2	0.68–40.7	3.0	0.39–23.3
Combined	134/32	1.4	0.70–2.9	3.9	1.4–10.9	2.2	0.79–6.3

N/E = amount of samples/events; *HR* = hazard ratio; 95%CI = 95% confidence interval; combined = combined estimates by the De Simmonian and Leird meta-analysis method. * Estimates adjusted by the overfitting bias according to Equation (3).

**Table 7:** Hazard ratios for the association between event-free survival of patients with Stage 4S neuroblastoma and *E2F3* gene expression, categorized on the basis of the median expression value and on the optimal cut-off.

		Cut-Off on Median Value		Optimal Cut-Off		Optimal Cut-Off (Adj) *	
Databases	N/E	*HR*	95%CI	*HR*	95%CI	*HR*	95%CI
Kocak	56/13	3.8	1.0–13.7	4.8	1.5–15.0	3.0	0.97–9.5
Oberthuer	30/7	6.8	0.81–56.2	10.0	1.9–52.5	7.9	1.5–41.7
SEQC	48/12	3.3	0.89–2.5	4.9	1.3–18.2	3.4	0.91–12.6
Combined	134/32	3.9	1.7–9.1	5.7	2.6–12.1	3.8	1.8–8.2

N/E = amount of samples/events; *HR* = hazard ratio; 95%CI = 95% confidence interval; combined = combined estimates by the De Simmonian and Leird meta-analysis method. * Estimates adjusted by the overfitting bias according to Equation (3).

## Data Availability

The data set used for the statistical validation (Section 3 and Section 4) and the databases used for the application of the proposed method (Section 5) are both included in Supplemental Material. They are also available on the Internet Platform AMC R2 (https://r2.amc.nl), accessed on 17 December 2019. Data from the immunofluorescence experiments are reported in Supplemental Table S1.

## Supplementary Materials

The following supporting information can be downloaded at: https://www.mdpi.com/article/10.3390/cancers15041188/s1, Figure S1: Theoretical Binormal distribution of gene expression levels assumed for the simulations under the alternative hypothesis of association between gene expression and patients survival. First group (Class 0, red line) with mean = µ_0_ and standard deviation = σ_0_ was associated with a constant event rate λ_0_; the second group (Class 1, green line) with mean = µ_1_ and standard deviation = σ_1_ was associated with a constant event rate λ_1_. *ODT* = Optimal decision threshold. Table S1: Diagnostic characteristics of Stage 4S NB patients whose tumours were analysed by immunofluorescence for *E2F1*; Table S2: Non-valid observations and outliers in estimates of the adjusted hazard ratio at an optimal cut-off by sample size in a set of 2000 simulated gene expression profiles. Two hidden normal distributions with an equal sample size were assumed with different means and equal variances. The first distribution was associated with an event rate = 0.1 (arbitrary units) and the second with a rate = 0.3 in an exponential survival model. Follow-up times have been right-censored at ten units; Table S3: Comparison between unadjusted and adjusted estimates of the hazard ratio at an optimal cut-off by sample size. Average of 2000 simulated values. Two hidden normal distributions with equal sample size were assumed with different means and equal variances. The first distribution was associated with an event rate = 0.1 (arbitrary units) and the second with a rate = 0.3, in an exponential survival model. Follow-up times have been right-censored at ten units. Estimates were obtained after exclusion of non-valid data and outliers; Table S4: Statistical power of the test associated with unadjusted and adjusted estimates of hazard ratio at an optimal cut-off at a nominal alpha level of 0.05 by sample size. Average of 2000 simulated values. Two hidden normal distributions with equal sample size were assumed with different means and equal variances. The first distribution was associated with an event rate = 0.1 (arbitrary units) and the second with a rate = 0.3, in an exponential survival model. Follow-up times have been right-censored at ten units. Estimates were obtained after exclusion of non-valid data and outliers, file: SupplementaryTables.pdf; Data used for statistical validation: Cangelosi et al. data set [9] including meta data and the first 1040 genes, file: DataSetCangelosi_1040.RData; Cavalli et al. data set [10], including meta data and the first 2000 gene expression profiles: DataSetCavalli_2000.RData; Results of statistical validation: *GenesName*: name of the gene in the original data sets; *MedVal*: median value in the training set; *lnHRMed*: Cox regression coefficient obtained using *MedVal* as a cut-off in the training set; *lnHRMedVar*: variance of *lnHRMed*; *lnHRMedAdj*: *lnHRMed* adjusted for the overestimation bias; *lnHRMedValid*: Cox regression coefficient obtained using *MedVal* as a cut-off in the test set; *OptVal*: optimal cut-off value in the training set; *lnHROpt*: Cox regression coefficient obtained using *OptVal* as a cut-off in the training set; *lnHROptVar*: variance of *lnHROpt*; *lnHROptAdj*: *lnHROpt* adjusted for the overestimation bias; *lnHROptValid*: Cox regression coefficient obtained using *OptVal* as a cut-off in the test set; files: StatisticalValidationResultsCangelosi.csv and StatisticalValidationResultsCavalli.csv; R script used for statistical validation on real datasets: HRValidCangelosi.R and HRValidCavalli.R; R scripts used for generation and statistical validation of simulated datasets, SimulAdjHRH0.R and SimulAdjHRH1.R; Demonstration of equation 4: DemonstrationEquation4.pdf. Data set used for the analysis described in Paragraph 4: NB4S.csv; R script for the analyses described in Paragraph 4: SurvNB4S.R.
