# Supplementary material for: A Simple, Test-Based Method to Control the Overestimation Bias in the Analysis of Potential Prognostic Tumour Markers
Source: Cancers (Basel). 2023 Feb 13;15(4):1188. doi: 10.3390/cancers15041188 (PMC9953998; doi:10.3390/cancers15041188)
Supplement: Supplementary file 1 [file cancers-15-01188-s001.zip › DemonstrationEquation (S4).pdf]

Demonstration of Equation (S4)

Let Class 0 and Class 1 be the distributions of a hypothetical gene expression in two groups of patients, each following a Normal probability distribution function (N_0 and N_1, respectively): $N_0 \sim N(\mu_0, \sigma^2_0)$ and $N_1 \sim N(\mu_1, \sigma^2_1)$ (Supplemental Figure S1). Let Class 0 and Class 1 be associated with an exponential survival probability with constant event rates λ_0 and λ_1 respectively. The Hazard Ratio HR_{opt} at the optimal discriminant threshold (*ODT*) can be obtained by the ratio of the corresponding event rates above and below *ODT* ($\bar{\lambda}_{>ODT}$ and $\bar{\lambda}_{<ODT}$, respectively):

$$HR_{opt} = \frac{\bar{\lambda}_{>ODT}}{\bar{\lambda}_{<ODT}} = \frac{\lambda_0 \int_{ODT}^{+\infty} N_0(x)\,dx + \lambda_1 \int_{ODT}^{+\infty} N_1(x)\,dx}{\lambda_0 \int_{-\infty}^{ODT} N_0(x)\,dx + \lambda_1 \int_{-\infty}^{ODT} N_1(x)\,dx} =$$

$$= \frac{\lambda_0 \left(1 - \int_{-\infty}^{ODT} N_0(x)\,dx\right) + \lambda_1 \left(1 - \int_{-\infty}^{ODT} N_1(x)\,dx\right)}{\lambda_0 \int_{-\infty}^{ODT} N_0(x)\,dx + \lambda_1 \int_{-\infty}^{ODT} N_1(x)\,dx} =$$

$$= \frac{\lambda_0 + \lambda_1}{\lambda_0 \int_{-\infty}^{ODT} N_0(x)\,dx + \lambda_1 \int_{-\infty}^{ODT} N_1(x)\,dx} - 1 =$$

$$= \frac{\lambda_0 + \lambda_1}{\lambda_0 \Phi\left(\frac{ODT - \mu_0}{\sigma_0}\right) + \lambda_1 \Phi\left(\frac{ODT - \mu_1}{\sigma_1}\right)} - 1$$

where Φ represents the standard normal cumulative distribution function.

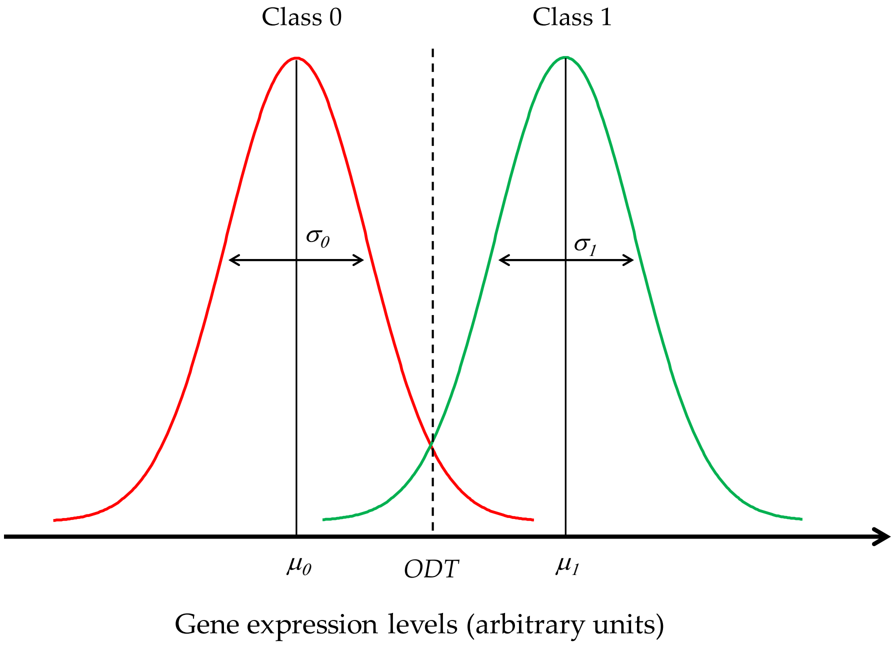

Supplemental Figure S1. Theoretical Binormal distribution of gene expression levels assumed for the simulations under the alternative hypothesis of association between gene expression and patients survival. First group (Class 0, red line) with mean = μ_0 and standard deviation = σ_0 was associated with a constant event rate λ_0; the second group (Class 1, green line) with mean = μ_1 and standard deviation = σ_1 was associated with a constant event rate λ_1. *ODT* = Optimal decision threshold.
