# Supplementary material for: A Simple, Test-Based Method to Control the Overestimation Bias in the Analysis of Potential Prognostic Tumour Markers
Source: Cancers (Basel). 2023 Feb 13;15(4):1188. doi: 10.3390/cancers15041188 (PMC9953998; doi:10.3390/cancers15041188)
Supplement: Supplementary file 1 [file cancers-15-01188-s001.zip › SupplementaryTables.pdf]

Ognibene et al.

A simple test-based method to control the overestimation bias in the analysis of potential prognostic tumour markers

Supplemental Table S1. Diagnostic characteristics of stage 4S NB patients whose tumours were analysed by immunofluorescence for *E2F1*.

Patient	INSS Stage	Protocol	*MYCN* amplified	Relapse	Relapse site	E2F1+ nuclei (%)
NB1	4S	Infant	No	No	-	4
NB2	4S	Infant	No	No	-	10
NB3	4S	Infant	No	No	-	7
NB4	4S	Infant	No	No	-	20
NB5	4S	Infant	No	No	-	6
NB6	4S	Infant	No	Yes*	bone marrow, liver	73
NB7	4S	Infant	Yes	Yes*	lung	75
NB8	4S	Infant	No	No	-	14
NB9	4S	Infant	No	No	-	7
NB10	4S	Infant	Yes	No	-	5
NB11	4S	Infant	No	No	-	2
NB12	4S	Infant	Yes	Yes	metastatic	68
NB13	4S	Infant	No	Yes	distant and local	71
NB14	4S	Infant	No	No	-	5
NB15	4S	Infant	No	No	-	9
NB16	4S	Infant	No	No	-	3
NB17	4S	Infant	No	No	-	14
NB18	4S	Infant	No	No	-	10
NB19	4S	Infant	No	No	-	4
NB20	4S	Infant	No	No	-	2
NB21	4S	Infant	No	No	-	6
NB22	4S	Infant	No	No	-	8
NB23	4S	Infant	No	No	-	7
NB24	4S	Infant	No	Yes	metastatic	74
NB25	4S	Infant	No	Yes	local	69
NB26	4S	Infant	No	Yes	metastatic	78
NB27	4S	Infant	No	No	-	7
NB28	4S	Infant	Yes	No	-	3
NB29	4S	Infant	No	No	-	11
NB30	4S	Infant	No	No	-	5
NB31	4S	Infant	No	No	-	2
NB32	4S	Infant	No	Yes	metastatic	76
NB33	4S	Infant	No	Yes	distant and local	64
NB34	4S	Infant	No	Yes*	central nervous system	77
NB35	4S	Infant	No	Yes	metastatic	71
NB36	4S	Infant	No	Yes	metastatic	62
NB37	4S	Infant	No	Yes	distant and local	73
NB38	4S	Infant	No	Yes*	bone marrow, bones	72

Y= yes; N= not; * tumor progression to true stage 4

Supplemental Table S2. Non-valid observations and outliers in estimates of adjusted Hazard Ratio at an optimal cut-off by sample size in a set of 2000 simulated gene expression profiles. Two hidden Normal distributions with equal sample size were assumed with different means and equal variances. The first distribution was associated with an event rate = 0.1 (arbitrary units) and the second with a rate = 0.3, in an exponential survival model. Follow-up times have been right-censored at ten units.

	Non valid estimates				Outliers			
	$\Delta\mu = 1$		$\Delta\mu = 5$		$\Delta\mu = 1$		$\Delta\mu = 5$	
Sample size	N	%	N	%	N	%	N	%
20*	25	1.3	162	8.2	153	7.7	200	10.1
40	6	0.3	17	0.9	116	5.8	63	3.2
80	15	0.8	5	0.3	50	2.5	4	0.2
120	9	0.5	1	0.05	39	2.0	1	0.05
160	16	0.8	0	0.0	38	1.9	0	0.0

Non valid estimates = adjusted HR with a bias higher than that observed iun the unadjusted ones. Outliers: HR estimates at the optimal cut-off more than three times higher than the true expected value (HR = 1.47 for $\Delta\mu = 1$, and HR = 2.95 for $\Delta\mu = 5$, respectively). $\Delta\mu$ = difference between the mean of the two hidden distributions.
* In the data set with $\Delta\mu = 1$, 11 observations without events and 1 very extreme outlier were excluded from the analyses. In the data set with $\Delta\mu = 5$, 15 observations without events and 9 very extreme outliers were excluded.

Supplemental Table S3. Comparison between unadjusted and adjusted estimates of Hazard Ratio at an optimal cut-off by sample size. Average of 2000 simulated values. Two hidden Normal distributions with equal sample size were assumed with different means and equal variances. The first distribution was associated with an event rate = 0.1 (arbitrary units) and the second with a rate = 0.3, in an exponential survival model. Follow-up times have been right-censored at ten units. Estimates were obtained after exclusion of non-valid data and outliers.

	$\Delta\mu = 1$			$\Delta\mu = 5$		
Sample size	HR_{un}	HR_{adj}	HR_{exp}	HR_{un}	HR_{adj}	HR_{exp}
20	1.90	1.57	1.47	3.64	3.00	2.95
40	2.23	1.64	1.47	3.87	2.96	2.95
80	2.28	1.69	1.47	3.45	2.54	2.95
120	2.16	1.66	1.47	3.27	2.24	2.95
160	2.07	1.63	1.47	3.18	2.02	2.95

HR_{un} = Unadjusted Hazard Ratio; HR_{adj} = Hazard Ratio adjusted for the overestimation bias; HR_{exp} = Expected Hazard Ratio at the optimal cut-off.

Supplemental Table S4. Statistical power of the test associated with unadjusted and adjusted estimates of Hazard Ratio at an optimal cut-off at 0.05 nominal alpha level by sample size. Average of 2000 simulated values. Two hidden Normal distributions with equal sample size were assumed with different means and equal variances. The first distribution was associated with an event rate = 0.1 (arbitrary units) and the second with a rate = 0.3, in an exponential survival model. Follow-up times have been right-censored at ten units. Estimates were obtained after exclusion of non-valid data and outliers.

	$\Delta\mu = 1$		$\Delta\mu = 5$	
Sample size	HR_{un}	HR_{adj}	HR_{un}	HR_{adj}
20	24.0%	11.0%	65.7	45.8
40	55.7%	20.5%	94.9	73.8
80	80.8%	34.9%	99.9	95.7
120	91.1%	47.0%	100	99.9
160	95.5%	58.5%	100	100

HR_{un} = inference on unadjusted esitimates of Hazard Ratio; HR_{adj} = inference on adjusted estimated of Hazard Ratio by permutation analysis.
